# Cell cycle dependent expression of the CCK2 receptor by gastrointestinal myofibroblasts: putative role in determining cell migration

**DOI:** 10.14814/phy2.13394

**Published:** 2017-10-16

**Authors:** Akos Varga, Jothi Dinesh Kumar, Alec W.M. Simpson, Steven Dodd, Peter Hegyi, Graham J. Dockray, Andrea Varro

**Affiliations:** ^1^ Department of Cellular and Molecular Physiology Institute of Translational Medicine University of Liverpool Liverpool United Kingdom; ^2^ First Department of Medicine University of Szeged Szeged Hungary; ^3^ Institute of Translational Medicine University of Pecs Pecs Hungary

**Keywords:** CCK2R, cell cycle, gastrin, migration, myofibroblasts

## Abstract

The well‐known action of the gastric hormone gastrin in stimulating gastric acid secretion is mediated by activation of cholecystokinin‐2 receptors (CCK2R). The latter are expressed by a variety of cell types suggesting that gastrin is implicated in multiple functions. During wound healing in the stomach CCK2R may be expressed by myofibroblasts. We have now characterized CCK2R expression in cultured myofibroblasts. Immunocytochemistry showed that a relatively small proportion (1–6%) of myofibroblasts expressed the receptor regardless of the region of the gut from which they were derived, or whether from cancer or control tissue. Activation of CCK2R by human heptadecapeptide gastrin (hG17) increased intracellular calcium concentrations in a small subset of myofibroblasts indicating the presence of a functional receptor. Unexpectedly, we found over 80% of cells expressing CCK2R were also labeled with 5‐ethynyl‐2′‐deoxyuridine (EdU) which is incorporated into DNA during S‐phase of the cell cycle. hG17 did not stimulate EdU incorporation but increased migration of both EdU‐labeled and unlabelled myofibroblasts; the migratory response was inhibited by a CCK2R antagonist and by an inhibitor of IGF receptor tyrosine kinase; hG17 also increased IGF‐2 transcript abundance. The data suggest myofibroblasts express CCK2R in a restricted period of the cell cycle during S‐phase, and that gastrin accelerates migration of these cells; it also stimulates migration of adjacent cells probably through paracrine release of IGF. Together with previous findings, the results raise the prospect that gastrin controls the position of dividing myofibroblasts which may be relevant in wound healing and cancer progression in the gastrointestinal tract.

## Introduction

In recent years myofibroblasts have emerged as important determinants of mucosal organization in health and disease (Ohlund et al. 2014). The role of these cells in wound healing in many different tissues is well‐known (Powell et al. 1999). In addition, however, a sheath of myofibroblasts lies just under the basement membrane in the gastrointestinal tract and is responsible for the secretion of proteins involved in extracellular matrix formation and turnover, as well as a range of growth factors including insulin‐like growth factors (IGF)‐1 and ‐2 (Hemers et al. 2005; Powell et al. 2005). In cancer, it is now clear that modified myofibroblasts (cancer‐associated myofibroblasts, CAMs) play a role in defining the cancer niche and in influencing cancer progression (De Wever et al. 2008). These cells may originate by epithelial‐mesenchymal transition (McCracken et al. 2014), from inward migration of bone‐marrow derived mesenchymal stem cells (Quante et al. 2011), or from tissue‐resident cells including fibroblasts and pericytes (Hosaka et al. 2016). The regulation of myofibroblast function remains incompletely understood.

The pyloric antral hormone gastrin plays a central role in regulating gastric acid secretion notably by stimulating synthesis and secretion of histamine from enterochromaffin‐like (ECL) cells which in turn stimulates acid secretion by parietal cells (Dockray 2004). In addition, gastrin regulates the growth of the oxyntic‐gland mucosa (Johnson 1976). This is most clearly seen in the hyperplasia of ECL cells that is associated with hypergastrinemia and which in extreme cases when accompanied by inflammation or mutations of the MEN‐1 gene may lead to neuroendocrine (carcinoid) tumors (Burkitt et al. 2009). These actions of gastrin are mediated by the cholecystokinin‐2 receptor (CCK2R) and are inhibited by CCK2R antagonists (Fossmark et al. 2012; Moore et al. 2013). In addition, there is growing recognition of the role of gastrin in gastrointestinal cancers including esophagus, stomach, pancreas, and colon (Ferrand and Wang 2006); in some cases the evidence points to a role for gastrin acting at CCK2R but there is also evidence of a role for nonclassical gastrins acting at other receptors (Kowalski‐Chauvel et al. 2012; Hayakawa et al. 2016).

The concept that tumors are wounds that do not heal is well recognized (Dvorak 1986; Desmouliere et al. 2004). In this context it is notable that expression of CCK2R occurs during wound healing in the stomach. Schmassmann and Reubi (2000) used in situ hybridization to show increased CCK2R in rat stomach following cryo‐ulceration; Ashurst et al. (2008) then showed that after cryo‐ulceration CCK2R expression was colocalized with *α*‐smooth muscle actin (*α*‐SMA) which is a biomarker for myofibroblasts (Desmouliere et al. 2004). The data therefore raise the possibility that CCK2R is expressed in activated myofibroblasts, but even so the significance of this is poorly understood. We now report that in many different gastrointestinal myofibroblasts there is transient expression of CCK2R in S‐phase of the cell cycle. We have tested the hypothesis that gastrin regulates migration of these cells in keeping with a role in determining cell position after exit from the cell cycle. The data suggest a novel dimension to understanding how gastrin might control gastric mucosal architecture.

## Materials and Methods

### Cells

Unless otherwise stated experiments were performed on human primary gastric CAMs previously generated from patients undergoing surgery for gastric cancer (Holmberg et al. 2012); some studies were also made on CAMs or myofibroblasts from tissue adjacent to cancers (ATMs) from colonic, pancreatic or esophageal cancer, normal tissue myofibroblasts (NTMs) from healthy stomach and esophagus, and myofibroblasts from chronic pancreatitis. The patients and the myofibroblasts obtained from them have all been described previously (Czepan et al. 2012; Holmberg et al. 2012; Kemeny et al. 2013; Kumar et al. 2014). The work was approved by the Ethics Committee of the University of Szeged, Szeged, Hungary and all subjects gave informed consent. Myofibroblasts were cultured as described previously and were used between passages 3 and 10 (Holmberg et al. 2012; Kemeny et al. 2013; Kumar et al. 2014). AGS cells were obtained from American Type Culture Collection (Manassas, VA). Wild type AGS cells were used together with stably transfected clones expressing CCK2R (Varro et al. 2002).

### Drugs, antibodies

L740093 was a gift from Dr R. Freidinger (Merck Sharpe and Dohme, Rathway, New Jersey), AG1024 was obtained from Calbiochem (Darmstadt, Germany), human unsulfated heptadecapeptide gastrin (hG17) from Bachem (St Helens, Merseyside, UK), Click‐iT EdU Imaging kit from Invitrogen (Paisley, UK), CCK2R antibody from Atlas Antibodies (HPA041976; Stockholm, Sweden), fluorescein (FITC)‐conjugated AffiniPure donkey anti‐rabbit IgG was from Jackson ImmunoResearch (West Grove, PA), Fluo‐4 AM and Pluronic F‐127 from Thermo Fisher Scientific (Waltham, MA) and other chemicals from Sigma‐Aldrich (Poole, Dorset, UK),

### Immunocytochemistry

Myofibroblasts were fixed with 4% w/v paraformaldehyde (PFA) and processed for immunocytochemistry as previously described (Wroblewski et al. 2003; Kumar et al. 2014). Cells were permeabilized with 0.2% Triton X‐100 in PBS followed by incubation with 5% w/v bovine serum albumin in PBS, and blocking with 10% v/v donkey serum. Myofibroblasts were stained with a CCK2R polyclonal antibody (1:200, overnight, 4°C) followed by incubation with FITC‐conjugated anti‐rabbit secondary antibody (1:400, 1 h, ambient temperature). In some experiments, cells were incubated in SF medium or hG17 (10 nmol/L, 24 h) and stained with an antibody to caspase‐3 (New England Biolabs, Hertfordshire, UK). Slides were mounted in Vectashield with 4′,6‐diamidino‐2‐phenylindole dihydrochloride (DAPI) and images were acquired using a Zeiss Axioplan‐2 fluorescence microscope (Zeiss Vision, Welwyn Garden City, UK). Images were recorded at 10× and 43× magnification using AxioVision software version 4.9.1.

### Microarray data

Microarray data on pairs of CAMs and corresponding ATMs from 13 gastric cancer patients have previously been deposited at http://www.ncbi.nlm.nih.gov/geo/query/acc.cgi?acc=GSE44740. The TNM classification system (Sobin et al. 2009) had been applied to these tumors which had shown that patients with early stage disease indicated by low lymph node involvement (pN0‐1) had significantly longer survival than those with advanced disease indicated by high lymph node involvement (pN2‐4) (51 vs. 12 months, respectively, *P* < 0.01) (Holmberg et al. 2012; Balabanova et al. 2014). The present analysis therefore focused on a comparison of CCK2R expression in these two groups. The abundance of CCK2R transcripts was expressed relative to GAPDH.

### Intracellular calcium

Subconfluent cells were loaded with Fluo‐4 AM ester (2 *μ*mol/L, 45 min, 37°C in HEPES saline buffer containing 10 mmol/L glucose, 1 mmol/L calcium, 200 *μ*mol/L sulphinpyrazone to reduce dye leakage and 25% w/v Pluronic F‐127 to increase cellular uptake of dye) as previously described (Homolya et al. 1993; Kao 1994). EGTA (1 mmol/L) was added to the solution to reduce spontaneous calcium activity. Myofibroblasts were stimulated with hG17 (10 nmol/L) or ionomycin (1 *μ*mol/L) as a positive control. Fluorescent signals were detected with a Leica SP2 AOBS multiphoton confocal microscope equipped with an argon‐ion laser at 488 nm absorbance and 516 nm emission peaks. Data analysis was carried out in LAS X 2.0 software.

### EdU incorporation

Proliferating cells were detected using 5‐ethynyl‐2′‐deoxyuridine (EdU) as previously described (Holmberg et al. 2012; Kumar et al. 2016). Myofibroblasts were synchronized by incubation in serum free (SF) media for 48 h followed by treatment with medium containing 10% fetal bovine serum (full medium, FM), or hG17, or fresh SF, and incubation with EdU reagent for up to 24 h. After EdU incorporation cells were fixed in paraformaldehyde and processed using Click‐iT^™^ Alexa Fluor 594 (300 *μ*L) according to the manufacturer's instructions. Staining of CCK2R was performed as described above.

### Flow cytometry

Cultured cells were incubated in SF or FM for 48 h, or were incubated in SF with or without hG17 (10 nmol/L, 24 h); cells were harvested and fixed in 4% PFA at 37°C for 10 min prior to permeabilization with 90% methanol for 30 min at 4°C. For cell cycle analysis, cells were directly stained with DAPI (1 *μ*g/mL). In experiments aimed at detecting CCK2R, myofibroblasts were washed twice with 0.5% BSA dissolved in 1xPBS and incubated with CCK2R primary antibody (1:200, 1 h, ambient temperature) and FITC‐conjugated secondary antibody (1:400, 30 min, ambient temperature) prior to nuclear staining with DAPI. Cells were sorted with FACS Canto II flow cytometer. CCK2R positive cells were separated based on fluorescence excited at 492 nm.

### Migration assays

Transwell migration and invasion assays were performed using BD inserts or BD BioCoat^™^ Matrigel^™^ invasion chambers (SLS, Nottingham, UK) with 1.3 or 2.5 × 10^4^ cells per insert, respectively, as previously described (Varro et al. 2004). Treatments including hG17 with or without L740093 or AG1024 added to the lower well. In some experiments EdU was added to the upper well and cells were incubated overnight. Membranes were then processed following the EdU protocol (see above) or alternatively cells were stained with Quick‐Diff (Reagena, Toivala, Finland).

### qPCR

RNA from control (SF) and gastrin (10 nmol/L, 24 h) treated myofibroblasts was extracted in Tri‐Reagent (1.25 mL; Sigma, Dorset, UK) according to the manufacturer's instructions. Pellets were resuspended in nuclease free water (50 *μ*L) and RNA (4 *μ*g) reverse transcribed with avian myeloblastosis virus reverse transcriptase and oligo‐dT primers (Promega). Real‐time PCR was carried out using an ABI7500 instrument (Applied Biosystems, Warrington, UK) and TaqMan primer/probe sets for human IGF‐1, IGF‐2 and GAPDH, together with Precision Plus 2× real‐time PCR master mix (Primer Design, Southampton, UK) and 5′‐FAM, 3′‐TAMRA double dye probes (Eurogentec, Southampton, UK). Values were standardized to GAPDH and assays included no‐template controls and a standard curve as previously described (Kumar et al. 2015). Primers and probes for detection of human IGF‐1, IGF‐2 and GAPDH, cDNAs were intron‐spanning and were: IGF‐1: 5′‐TGT ATT GCG CAC CCC TCA A‐3′ (forward), 5′‐CT CCC TCT ACT TGC GTT CTT CA‐3′ (reverse), 5′‐ACA TGC CCA AGA CCC AGA AGG AAG TAC A‐3′ (probe); IGF‐2, 5′‐CCG TGC TTC CGG ACA ACT T‐3′ (forward), 5′‐GGA CTG CTT CCA GGT GTC ATA TT‐3′ (reverse), 5′‐CCC AGA TAC CCC GTG GGC AAG TTC‐3′ (probe); GAPDH: 5′‐GCT CCT CCT GTT CGA CAG TCA‐3′(forward), 5′‐ACC TTC CCC ATG GTG TCT GA‐3′ (reverse), 5′‐CGT CGC CAG CCG AGC CAC A‐3′ (probe).

### Statistics

Results are expressed as means ± S.E. and comparisons were made by ANOVA, t test or Fisher exact test, as appropriate.

## Results

### CCK2R is expressed in a subset of myofibroblasts

**Figure 1:**
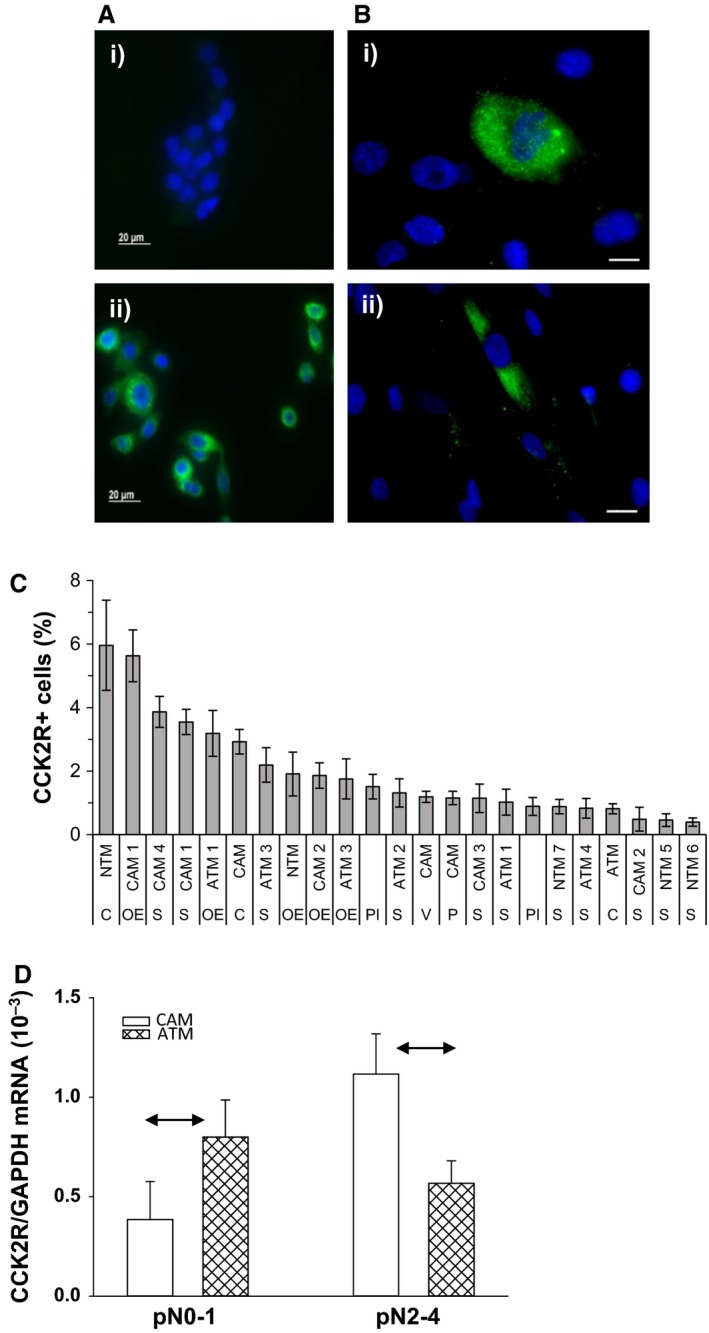
Localization of CCK2R to a subset of myofibroblasts. (A) *V*alidation of antibody specificity: i) in wild type AGS cells, which do not express CCK2R, there is no signal compared with ii) a strong CCK2R signal in AGS‐Gr cells which have been stably transfected with a receptor‐encoding construct (nuclei stained blue with DAPI). (B) Myofibroblasts from several regions of the gastrointestinal tract express CCK2R in a subset of cells; i), gastric cancer associated myofibroblasts (CAMs); ii), normal stomach myofibroblasts. (C) Expression in myofibroblasts from different regions of the gut (C, colon; OE, esophagus; S, stomach; PI, chronic pancreatitis; P, pancreatic cancer; ATM, adjacent tissue to cancer; NTM, normal tissue myofibroblasts). (D) Abundance of CCK2R transcripts relative to GAPDH in 13 pairs of CAMs and their corresponding ATMs from gastric cancer patients, derived from microarray data. The patients are divided into two groups depending on lymph node involvement (pN0‐1, *n* = 7; pN2‐4, *n* = 6). Means ± SE, horizontal arrows significantly different *P* < 0.05.

In initial experiments we validated the antibody used for immunocytochemical localization of CCK2R by comparison of wild type AGS cells (which do not express the receptor) with their counterparts that have been stably transfected with CCK2R, i.e AGS‐Gr cells (Varro et al. 2002). As expected, the parental cell line was not stained; however, AGS‐Gr cells were strongly positive (Fig. 1A). We then examined expression of CCK2R in a number of myofibroblast populations recovered from normal tissue, cancer or cancer‐adjacent tissue from esophagus, stomach or colon, or pancreatitis. In all cases, only a subpopulation of cells were positive for CCK2R, ranging from 1 to 6% (Fig. 1B and C). We next examined the expression of CCK2R in a microarray dataset (http://www.ncbi.nlm.nih.gov/geo/query/acc.cgi?acc=GSE44740) of CAMs and ATMs prepared from 13 patients with gastric cancer (Balabanova et al. 2014). Interestingly, in patients with high lymph node involvement (graded pN2‐4), which correlates with poor postoperative survival, the expression of CCK2R was higher in 5 of 6 CAMs compared with their matched ATMs, whereas in the subgroup with low lymph node involvement (pN0‐1) 6 of 7 CAMs expressed lower CCK2R compared with their matched ATMs (Fig. 1D) and the difference was statistically significant (*P* < 0.05, Fisher exact test). Although the numbers are smaller, the same picture can be seen in the immunocytochemical data shown in Figure 1C where expression of CCK2R was higher in CAMs compared with their matched ATMs from patients with high lymph node involvement (S‐CAM4/ATM4, pN2; S‐CAM1/ATM1, pN2) while the converse is true for patients with low lymph node involvement (S‐CAM2/ATM2, pN0; S‐CAM3/ATM3, pN1).

### Gastrin increases intracellular calcium in a subset of myofibroblasts

**Figure 2:**
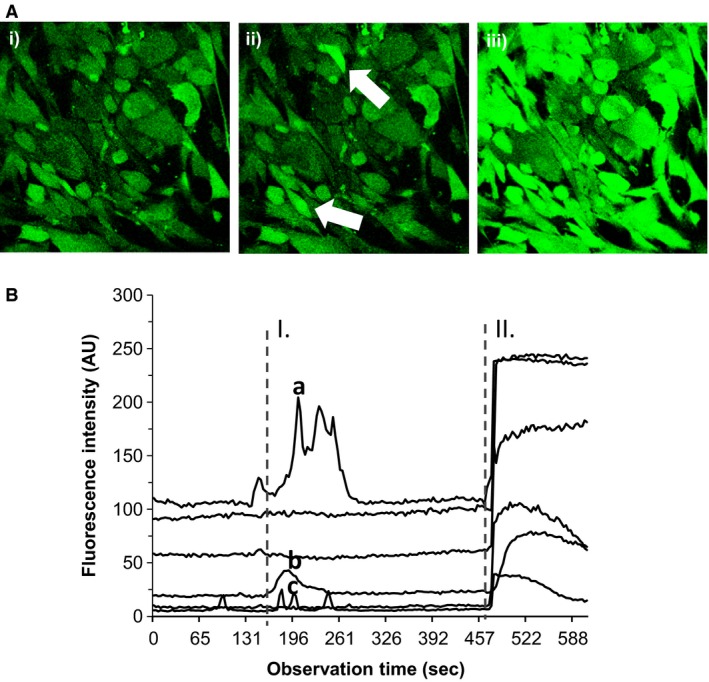
Gastrin increases intracellular calcium in a subset of myofibroblasts. (A) Labeling of gastric CAMs with Fluo‐4; i), control, ii) stimulation with hG17 (10 nmol/L), arrows indicate responding cells; iii) effect of ionomycin (1 *μ*mol/L). (B) hG17 (applied at I) stimulated a transient increase in intracellular calcium in a subpopulation of gastric CAMs (a, b, c); ionomycin (applied at II) produced a sustained increase in calcium in the majority of cells.

In order to determine whether myofibroblasts are capable of mounting a functional response to gastrin we then examined changes in intracellular calcium on administration of hG17. In myofibroblasts in SF medium, there was labeling with Fluo‐4 and no or infrequent spontaneous fluctuations in intracellular calcium. Administration of hG17 (10 nmol/L) produced a prompt increase in intracellular calcium in a subset of gastric CAMs (5.3 ± 1.4% of total; 169 cells counted in three fields (Fig. 2A and B). When the calcium ionophore was administered there was a sustained increase in intracellular calcium in virtually all cells.

### CCK2R expression is associated with the cell cycle

**Figure 3:**
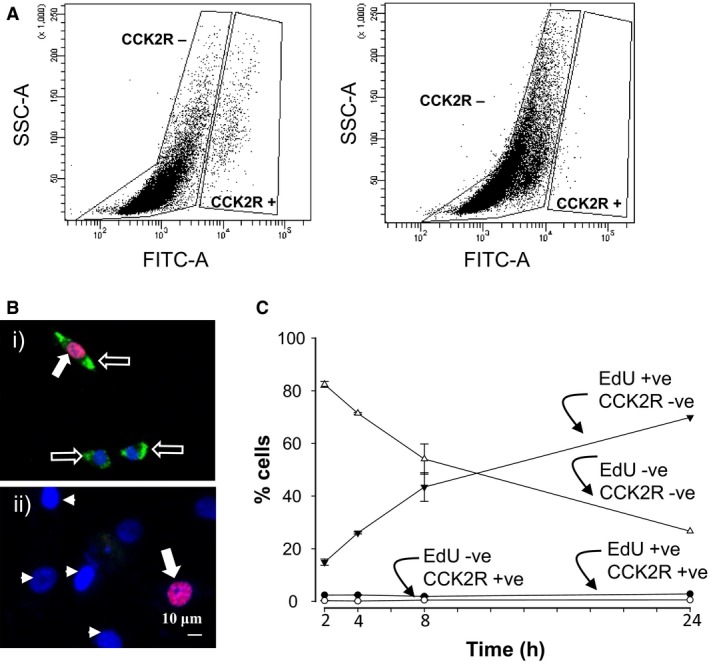
CCK2R expression is associated with proliferation. (A) Flow cytometry shows a subpopulation of cells cultured in full medium express CCK2R (left) but this population is diminished by incubation of cells in serum free medium (right) which suppresses proliferation. (B) CCK2R is expressed in cells that incorporate EdU; i), a gastric CAM incorporating EdU (red nuclei) expresses CCK2R (green) but there are also EdU‐negative cells expressing CCK2R; ii), an example of a gastric CAM exhibiting EdU incorporation but not CCK2R expression; filled arrows, EdU labeled nuclei; open arrows, CCK2R; arrow heads, DAPI‐stained nuclei in EdU‐ and CCK2R‐negative cells. (C) Incorporation of EdU increases with time (▼), but the proportion of cells expressing CCK2R and labeled with EdU (●) remains relatively constant at 2–3% total; less than 1% of cells express CCK2R and are not labeled with EdU (○), a decreasing proportion of cells are both EdU and CCK2R negative (Δ). Means ± SE (*n* = 3).

To determine whether CCK2R expression was influenced by culture conditions, we incubated gastric CAMs in FM or SF medium and examined expression by flow cytometry. The subset of cells expressing CCK2R amounted to approximately 2% of total cells in FM, but when cells were cultured in SF medium to depress proliferation, the population of CCK2R‐labelled cells was approximately 0.6% total (Fig. 3A). We then considered the hypothesis that expression of CCK2R was dependent on the cell cycle. As expected, cells incubated in FM exhibited significantly higher labeling with EdU (which is incorporated into DNA in S‐phase) compared with cells in SF medium (28.7 ± 0.3 vs. 5.2 ± 0.6% cells incorporating EdU, respectively, *P* < 0.001). Interestingly, when we double‐labeled cells with EdU and CCK2R (Fig. 3B), over 80% of cells expressing CCK2R were found to be labeled with EdU. However, while the proportion of cells showing EdU incorporation increased with duration of incubation in FM the proportion of cells expressing CCK2R remained relatively constant at 2–3% of total; similarly the subpopulation of CCK2R positive cells that were not labeled with EdU (<1% of total) remained relatively constant (Fig. 3C).

### Gastrin does not stimulate proliferation or apoptosis of myofibroblasts

**Figure 4:**
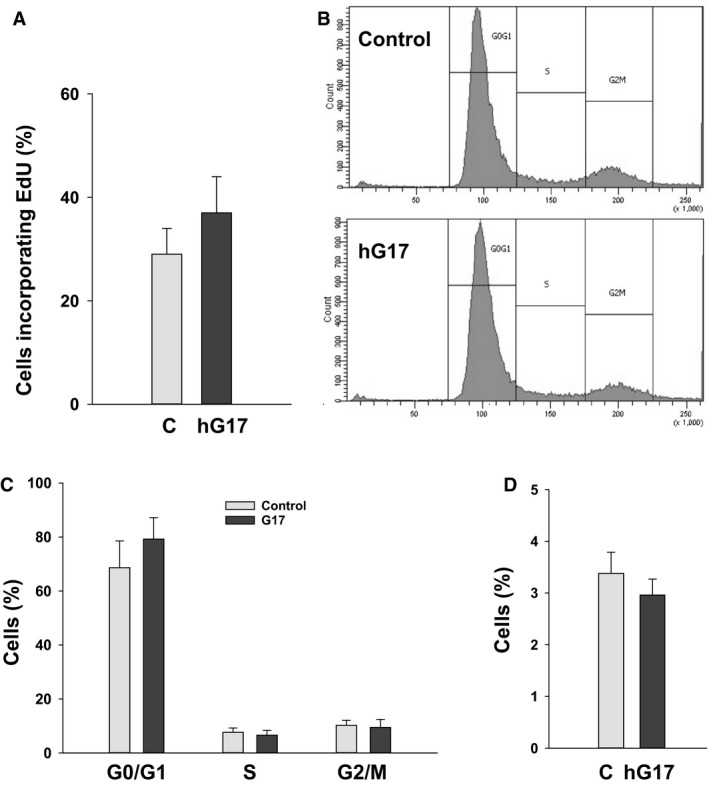
Gastrin does not stimulate proliferation or apoptosis in myofibroblasts. (A) Incubation in hG17 (10 nmol/L, 24 h) had no effect on EdU incorporation. (B) Representative flow cytometry profiles show no effect of incubation in hG17 on the proportion of cells in G0/G1, S or G2/M. (C) Quantification of cells in G0/G1, S or G2/M by flow cytometry in three separate experiments. (D) Staining with antibody to caspase‐3 shows no effect of hG17 (10 nmol/L, 24 h) on apoptosis. *N* = 3, means ± SE.

Since CCK2R expression was associated with EdU incorporation we then asked whether gastrin influenced EdU incorporation. We found no significant difference in EdU incorporation in response to gastrin (Fig. 4A). Moreover in flow cytometry, the proportions of cells in G0/G1, S or G2/M phases of the cell cycle were not significantly different after incubation with hG17 (Fig. 4B and C). Gastrin also had no significant effect on caspase‐3 labeling used as a marker of apoptosis (Fig. 4D).

### Gastrin stimulates migration and invasion of EdU‐labeled and unlabeled gastric myofibroblasts

**Figure 5:**
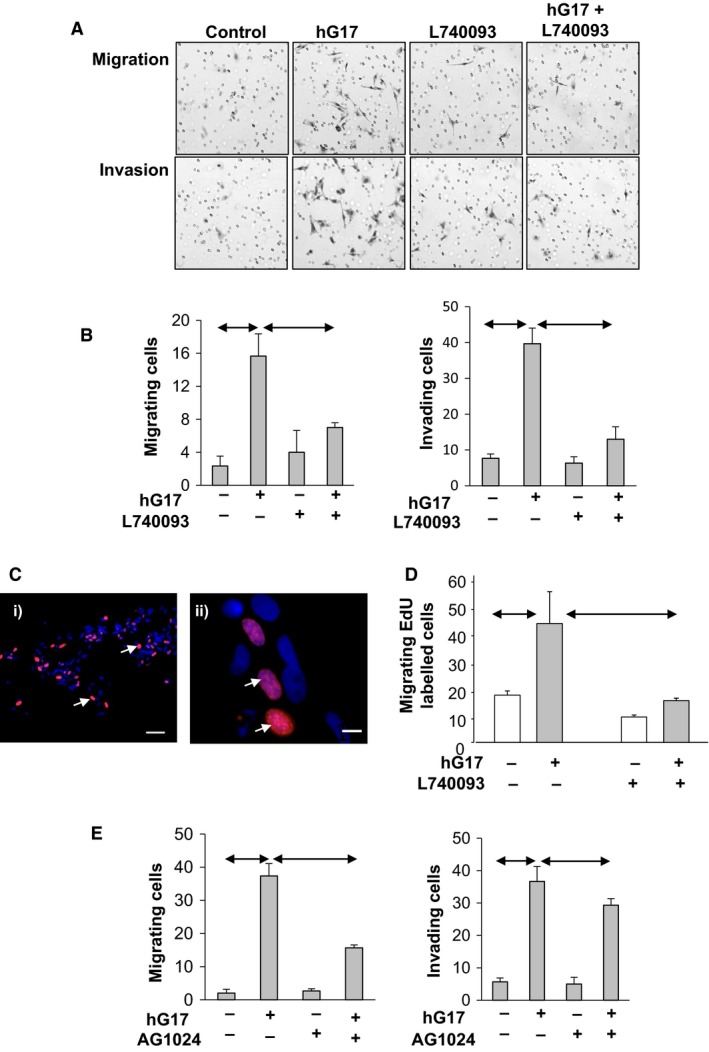
Gastrin stimulates myofibroblast migration and invasion. (A) Representative Boyden chamber images showing migration (top) and invasion (bottom) in response to hG17 (10 nmol/L), L740093 (50 *μ*mol/L), and the combination. (B) hG17 stimulates both cell migration (left) and invasion (right) of myofibroblasts in Boyden chambers and the response is inhibited by the CCK2R antagonist, L740093; *y*‐axis mean ± SE number of cells per field. (C) Some migrating cells incorporate EdU (blue, DAPI; red, EdU, arrows); i) scale bar 50 *μ*m, ii) 10 *μ*m. (D) hG17 stimulates migration of EdU labeled cells and this is blocked by L740093 (*y*‐axis labeled cells per field). (E) Migration (left) and invasion (right) in response to hG17 was suppressed by the IGF‐1 receptor tyrosine kinase inhibitor (AG1024, 2 *μ*mol/L). Horizontal arrows, *P* < 0.05, ANOVA.

Since stimulation of myofibroblasts is linked to migration and invasion, we then examined the effect of hG17 on these responses using Boyden chambers. There was a clear stimulation of both migration and invasion by hG17 that was inhibited by the CCK2R antagonist L740093 (Fig. 5A and B). To further characterize the migrating cells we examined whether they incorporated EdU. There was indeed hG17‐stimulated migration of EdU labeled cells that was inhibited by L740093 (Fig. 5C and D). However, only about 40% of the migrating cells were labeled with EdU suggesting that cells expressing CCK2R were also able to activate other cells via a paracrine pathway. A role for IGF in mediating autocrine/paracrine signaling in myofibroblasts is well‐established (Hemers et al. 2005; McCaig et al. 2006), and consistent with this we found the IGF receptor tyrosine kinase inhibitor AG1024 suppressed hG17‐stimulated migration, and to a lesser extent invasion (Fig. 5E). Finally, in the presence of hG17 the relative abundance of IGF‐2 transcripts was 2.1 ± 0.1 fold higher than control (*P* < 0.05); IGF‐1 transcript abundance was virtually undetectable in these cells.

## Discussion

In the normal gastrointestinal tract, CCK2R expression is localized to parietal cells, ECL cells, an LGR5 +  antral stem cell population, smooth muscle and some enteric neurons (Dufresne et al. 2006; Hayakawa et al. 2016). However, in wound healing there is induction of CCK2R in cells of a fibroblastic lineage which may include myofibroblasts (Schmassmann and Reubi 2000; Ashurst et al. 2008). The main finding of the present study is that CCK2R is expressed in myofibroblasts from different parts of the gut while the cells are in S‐phase of the cell cycle. The receptor appears to be functional and stimulation is associated with increased intracellular calcium and increased migration and invasion. Taken together it appears that expression of CCK2R may provide a mechanism that allows gastrin to determine the position of myofibroblasts as they progress through the cell cycle which might be particularly relevant during wound healing or in cancer.

There are well‐known issues regarding interpretation of immunocytochemical studies with antibodies to G‐protein coupled receptors (GPCRs) (Baker 2015). The antibody we employed was validated by the finding that wild type AGS cells (which do not express the receptor) were unstained while there was a strong signal in those cells that were stably transfected with CCK2R. Similar results (not shown) have also been obtained with an esophageal cell line (OE33 cells) transfected with the receptor (Haigh et al. 2003). These findings therefore provide direct support for the specificity of the antibody in localizing CCK2R.

The expression of CCK2R varied from 1 to 6% of cells in different myofibroblast lines. However, expression of CCK2R was higher in CAMs compared with their corresponding ATMs in patients with advanced gastric cancer and in these circumstances CAMs are known to have higher rates of proliferation (Holmberg et al. 2012), moreover a high proportion of CCK2R expressing cells incorporated EdU which corresponds to S‐phase of the cell cycle. Although the proportion of CCK2R‐expressing cells appears relatively low, it is similar to the proportion determined to be in S‐phase from flow cytometry. Because EdU incorporation into DNA is stable, labeled cells correspond to those that were in S‐phase at some stage during the labeling period. This is likely to account for the observation that while most CCK2R‐expressing cells were also labeled with EdU, there were nevertheless populations of EdU labeled cells that were CCK2R negative (i.e corresponded to cells that had since left S‐phase and ceased expressing the receptor). The data indicate, therefore, that CCK2R expression in myofibroblasts is transiently associated with S‐phase although in a formal sense we cannot exclude the possibility that there are two distinct populations of cells, one of which expresses CCK2R during S‐phase and the other which does not. However, while a relatively small proportion of cells may be expressing CCK2R at any one point in time, expression restricted to S‐phase means that all cells in this population do indeed express CCK2R, albeit transiently. Thus looking at overall frequency of expression at a single time point may be misleading.

The GPCR agonists that are linked to increases in intracellular calcium are also mitogens for many different cell types where proliferative responses may reflect transactivation of the EGF receptor (Prenzel et al. 1999; Varro et al. 2002). In cells of fibroblastic lineages there is an association among cell shape, cell cycle progression, and calcium influx (Pennington et al. 2007). For the most part the focus of previous work in this area has been on progression from the checkpoint to S‐phase. The selective expression of GPCRs that serve to increase intracellular calcium only in S‐phase suggests mechanisms that are more complex than previously supposed. Our observations suggest that gastrin does not influence either EdU incorporation during S‐phase or progression through G‐2 to mitosis. This is interesting not least because the situation appears to be different for some other GPCRs that exhibit cell‐cycle dependent regulation of expression. For example the chemokine receptor CXCR3 is expressed in both S and G2‐M phases in human microvascular endothelial cells (Romagnani et al. 2001); moreover, expression of GPR19 in lung cancer cells is triggered by entry into S‐phase and in this case seems to promote progression from G2 to M phase (Kastner et al. 2012).

The capacity of CCK2R/EdU labeled cells to respond to gastrin in chemotaxis assays provides direct evidence that the receptor is linked to a functional response. Interestingly, in these experiments there was also increased migration of unlabelled cells. As already mentioned, it is established that gastrin activates a number of paracrine signaling cascades in epithelial cells including EGF‐family members, FGF‐2, IL‐8, and prostaglandins (Varro et al. 2002, 2004; Noble et al. 2003). The present data suggest gastrin may also target the IGF system which is important for mediating paracrine mechanisms initiated by gastrointestinal myofibroblasts (Hemers et al. 2005; McCaig et al. 2006). Thus, we show here that the IGF receptor tyrosine kinase inhibitor blocked the paracrine effect of gastrin on migration of unlabelled cells, moreover gastrin increased IGF‐2 transcript abundance. Whether there are other mediators requires further work.

In experimental studies in rat, CCK2R is expressed during wound healing in the stomach (Schmassmann and Reubi 2000); in addition to epithelial cell expression the evidence indicates that several days after injury CCK2R is expressed in submucosal cells that express the myofibroblast marker, *α*‐SMA (Ashurst et al. 2008). The mechanisms regulating expression have been studied in cultured epithelial cells where it has been shown that serum starvation and gastrin itself increase CCK2R expression (Ashurst et al. 2008). It now seems likely that different mechanisms regulate CCK2R expression in different cell types and at present it would appear that the association between expression and S‐phase of the cell cycle is a property of myofibroblasts. Given the importance of myofibroblasts in tissue repair the expression of CCK2R may well underpin the gastrin‐stimulated wound healing observed in animal models (Schmassmann and Reubi 2000).

There has been growing interest in the role of gastrin in promoting cancer progression (Ferrand and Wang 2006; Kovac et al. 2009; Boyce et al. 2017). In part, this work has been directed at the nonclassical gastrins that are thought not to act at CCK2R. The situation is not clear cut, however, since there may be a role for CCK2R in mediating the effects of progastrin (which is not a classical CCK2R agonist) on colon cancer progression (Jin et al. 2009). To the extent that research in this area has been directed at CCK2R expression it has generally been focused on receptor expression by either cancer cells or normal epithelial cells, including stem cells. The idea that CCK2R might also be transiently expressed in myofibroblasts adds a new dimension, which is of particular interest in view of the emerging consensus that the microenvironment is an important determinant of cancer progression (De Wever et al. 2008; Hanahan and Weinberg 2011; Quail and Joyce 2013).

## Conflict of Interest

The authors disclose no conflicts of interest, financial or otherwise.
